# Fe_3_O_4_@MOF-808磁性固相萃取结合高效液相色谱法测定大米中3种二苯醚类除草剂

**DOI:** 10.3724/SP.J.1123.2020.06007

**Published:** 2021-03-08

**Authors:** Meng YAN, Yeqing JIA, Peiru QI, Yahui WANG, Qianqian JI, Manman WANG, Qian WANG, Yulan HAO

**Affiliations:** 华北理工大学公共卫生学院, 河北 唐山 063210; School of Public Health, North China University of Science and Technology, Tangshan 063210, China; 华北理工大学公共卫生学院, 河北 唐山 063210; School of Public Health, North China University of Science and Technology, Tangshan 063210, China; 华北理工大学公共卫生学院, 河北 唐山 063210; School of Public Health, North China University of Science and Technology, Tangshan 063210, China; 华北理工大学公共卫生学院, 河北 唐山 063210; School of Public Health, North China University of Science and Technology, Tangshan 063210, China; 华北理工大学公共卫生学院, 河北 唐山 063210; School of Public Health, North China University of Science and Technology, Tangshan 063210, China; 华北理工大学公共卫生学院, 河北 唐山 063210; School of Public Health, North China University of Science and Technology, Tangshan 063210, China; 华北理工大学公共卫生学院, 河北 唐山 063210; School of Public Health, North China University of Science and Technology, Tangshan 063210, China; 华北理工大学公共卫生学院, 河北 唐山 063210; School of Public Health, North China University of Science and Technology, Tangshan 063210, China

**Keywords:** 磁性固相萃取, 高效液相色谱, 二苯醚类除草剂, 大米, 金属有机骨架, magnetic solid phase extraction (MSPE), high performance liquid chromatography (HPLC), diphenyl ether herbicides (Des), rice, metal organic frameworks (MOFs)

## Abstract

利用溶剂热法构筑了Fe_3_O_4_@MOF-808吸附剂,将其用于大米中除草醚(NIT)、乙氧氟草醚(OXY)和甲羧除草醚(BIF)3种二苯醚类除草剂的富集,结合高效液相色谱法,建立了大米中该类除草剂的分析方法。研究通过傅里叶变换红外光谱、X射线衍射仪、扫描电子显微镜以及振动样品磁强计对构筑的磁性吸附剂的结构、表面形貌及磁强度进行表征。表征结果显示,球形的Fe_3_O_4_纳米颗粒与八面体形貌的MOF-808成功复合,Fe_3_O_4_@MOF-808饱和磁化强度可达40.35 emu/g,可以满足磁性固相萃取的需求;对吸附剂用于大米中3种二苯醚类除草剂富集的磁性固相萃取条件(吸附剂用量、吸附时间、洗脱溶剂种类以及洗脱体积)进行了优化。优化结果显示,25 mg吸附剂在6 min内即可达到对目标物的完全吸附,洗脱溶剂采用0.5 mL×2的甲醇。在最优的磁性固相萃取条件下,结合高效液相色谱-紫外检测法,建立了大米中3种二苯醚类除草剂的分析方法。方法在2~300 μg/L范围内线性关系良好(*r* > 0.998), NIT、OXY、BIF的检出限和定量限依次为0.6、0.6、0.4 μg/kg和2.0、2.0、1.5 μg/kg,在5、10和20 μg/kg 3个加标水平下的回收率为87.3%~96.7%,相对标准偏差不超过10.8%,且富集因子在25~29之间。将所建方法用于大米中NIT、OXY、BIF的分析,各样品均未检出这3种二苯醚类除草剂。该方法具有操作简单、快速、准确的特点,适用于大米样品中除草剂的残留分析。

## Abstract (en)

The complex matrix of rice samples and the small amount of the target analytes in the sample necessitate an effective pretreatment process to enrich the target analytes and minimize matrix interference before instrumental analysis. Magnetic solid phase extraction (MSPE) is a dispersive solid phase extraction technique which allows for the rapid separation of sorbents from the sample solution under an external magnetic field. Compared with other traditional solid phase extraction methods, MSPE has the advantages of convenient operation, minimal interference and absence of column pressure. In this work, a metal organic framework composite (Fe_3_O_4_@MOF-808) was synthesized by a facile solvothermal method for using as an effective adsorbent to concentrate nitrofen (NIT), oxyfluorfen (OXY) and bifenox (BIF) in rice samples. Based on the pretreatment, a method was developed by coupling with high performance liquid chromatography-ultraviolet detection (HPLC-UV). The prepared material was characterized by Fourier-transform infrared spectroscopy, X-ray diffractometry, scanning electron microscopy and vibrating sample magnetometry measurements for determining its functional groups, morphology and magnetic strength. The results showed that MOF-808 has a regular octahedral morphology and well-dispersed, and the particle size of the material ranged from 400 to 500 nm with a smooth surface. The spherical Fe_3_O_4_ particles were uniformly attached to the surface of the octahedral MOF-808 crystals. The maximum saturation magnetization of this composite was 40.35 emu/g which is lower than the saturation magnetization Fe_3_O_4_ (78.26 emu/g) but still sufficient for the requirements of MSPE. The prepared Fe_3_O_4_@MOF-808 was used in the MSPE of three diphenyl ether herbicides (Des) in rice. As is well known, the key factors influencing MSPE are the adsorption and elution processes. In order to establish the optimal extraction conditions, the adsorption parameters (adsorbent amount, extraction time, elution solvent and elution volume) were investigated in detail. A 15 mL mixed standard solution was used in the experiment, and the concentrations of the three Des were 65 ng/mL. All the experiments were performed in parallel three times. The effects of the dosages of Fe_3_O_4_@MOF-808 (10, 15, 20, 25 and 30 mg), adsorption time (2, 4, 6, 8 and 10 min), elution solvents (acetone, acetonitrile and methanol) and elution volume (0.5 mL, 0.5 mL×2, 0.5 mL×3, 0.5 mL×4) were investigated. The Des could be adsorbed completely by using 25 mg of Fe_3_O_4_@MOF-808 for no more than 6 min. Elution was performed with 0.5 mL×2 of methanol. Various parameters such as limits of detection (LODs), limits of quantification (LOQs), accuracy and precision of the method were evaluated. The method showed good linearity in the range of 2-300 μg/L (*r* > 0.998). The LODs and LOQs were 0.6, 0.6, 0.4 μg/kg and 2.0, 2.0,1.5 μg/kg for NIT, OXY, BIF respectively. At spiked levels of 5, 10 and 20 μg/kg, the recoveries ranged from 87.3% to 96.7% with relative standard deviations (RSDs) less than 10.8%. The enrichment factors (EFs) of the method for the three Des were between 25 and 29. The method was applied to the pretreatment of the three Des in real samples, and none of the Des could be detected at any of the samples. This method had a lower LOD than that of the national standard method, but its LOD and recovery were similar to those of other reference methods. In summary, the developed method has the advantages of operational simplicity, rapidity and accuracy, and it is suitable for the analysis of herbicide residues in rice samples.

二苯醚类除草剂(diphenyl ether herbicides, Des)是一种原卟啉原氧化酶抑制剂,主要用于防除一年生和多年生阔叶杂草,是水稻田主要的除草剂品种之一^[1]^。Des在环境中具有累积性和持久性,对生物体的影响不容忽视。研究表明,大部分Des会对眼睛和皮肤产生刺激作用^[2]^。除草醚可以引起高铁血红蛋白血症、溶血性贫血和黄疸等疾病;甲羧除草醚对鱼及水生动物具有高毒作用。常见的Des有除草醚(nitrofen, NIT)、乙氧氟草醚(oxyfluorfen, OXY)和甲羧除草醚(bifenox, BIF),其结构式见图1。国标GB 2763-2019^[3]^对水稻种植过程中Des的使用及大米中Des的残留限值做出了规定,规定糙米中OXY的最大残留限量为0.05 mg/kg。因此,及时掌握大米中的Des残留情况,建立快速、可靠的大米中Des检测方法具有重要意义。

**图1:**
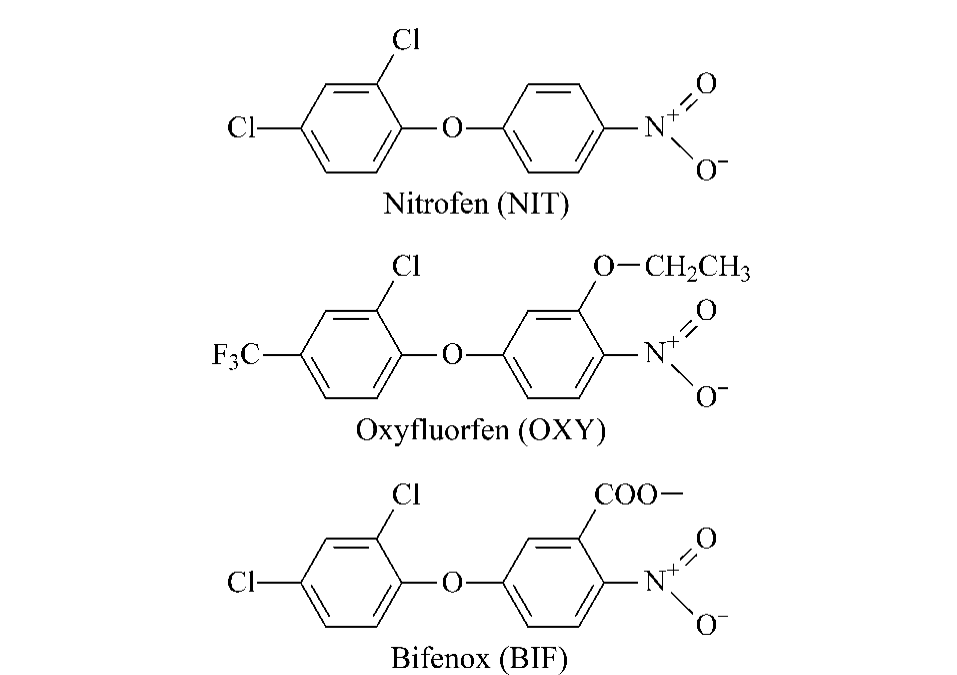
3种二苯醚类除草剂的化学结构式

目前,常采用高效液相色谱法(HPLC)、气相色谱法和气相色谱-质谱法等分析谷物中的Des^[4,5,6]^。由于大米样品基质复杂,且目标分析物在样品中的含量较低,不易检出。在仪器分析前,需要对样品进行充分净化以去除杂质干扰,同时对目标物进行富集。大米样品中除草剂残留的前处理方法主要有固相萃取和分散固相萃取^[4,7]^。磁性固相萃取(magnetic solid phase extraction, MSPE)属于分散固相萃取技术中的一种,其通过施加外部磁场可以实现吸附剂与样品溶液的快速分离^[8]^。与传统填充柱相比,MSPE具有操作步骤简便、基质干扰小、无需填充萃取柱以及避免柱压力等优点^[9]^。MSPE吸附剂是由磁性核心和具备吸附作用的吸附材料组成,其中磁性核心一般采用Fe_3_O_4_,具备吸附作用的有机基团可以根据待测物进行灵活选择。吸附材料是决定磁性吸附效果的关键。

金属-有机骨架(metal organic frameworks, MOFs)是以有机配体中的氧原子和氮原子连接无机金属离子形成的多孔晶体材料,具有比表面积大、溶剂稳定性好以及孔道可调控等特性而备受关注^[10]^。以Zr金属为中心的MOFs具有良好的热稳定性与化学稳定性,是近年来发展迅速的一种新型吸附剂。MOF-808由氯化锆和1,3,5-苯三甲酸制备而成,以Zr_6_O_4_(OH)_4_(-CO_2_)_6_(HCOO)_6_为二级构建单元,与6个1,3,5-苯三甲酸单元连接,通过桥连作用形成超四面体结构^[11]^。该四面体笼的内孔尺寸为0.48 nm,由10个连续的四面体笼形成大金刚烷笼的内部孔径为1.84 nm,比表面积可以达到2060 $m^{2}/g^{}$ ^[12]^。同时,MOF-808结构中丰富的金属位点及有机配体可以提供*π-π*共轭、氢键作用,使其具备良好的吸附分离功能^[13,14]^。

本研究利用溶剂热法将磁性核心Fe_3_O_4_与MOF-808进行组装,构筑Fe_3_O_4_@MOF-808吸附剂,可同时发挥MSPE技术优势及MOF-808的优良性能。将其用于大米中3种Des的前处理,结合HPLC-UV,建立了简单、快速测定大米中3种Des的分析方法。

## 1 实验部分

### 1.1 仪器和试剂

Agilent 1200型高效液相色谱-紫外检测器(美国Agilent公司); JEM-2800F聚焦离子束-电子束双束电镜(美国FEI仪器有限公司); FTIR-8400S傅里叶变换红外光谱仪(日本Shimadzu公司); D8-Venture单晶X射线衍射仪(德国布鲁克公司); 9600-1振动样品磁强计(美国LDJ公司); 100 mL-水热合成反应釜(天津凯易达仪器设备销售有限公司); ET-3301A型氮气浓缩装置(上海欧陆科仪有限公司)。

1,3,5-苯三甲酸(H_3_BTC)、氯化锆(ZrCl_4_)、*N*,*N*'-二甲基甲酰胺(DMF)、甲酸和乙二醇(纯度99%,上海阿拉丁试剂有限公司);六水合氯化铁(FeCl_3_·6H_2_O)、三水合乙酸钠(NaOAc·3H_2_O)和无水乙醇(纯度99%,天津光复科技有限公司);甲醇(methanol, MeOH)和乙腈(acetonitrile, ACN)(色谱纯,美国Thermo Fisher Scientific公司);娃哈哈纯净水(杭州娃哈哈集团有限公司)。

标准物质NIT(纯度99.0%)和OXY(纯度99.5%)(上海阿拉丁试剂有限公司); MeOH配制的100 μg/mL BIF(纯度99.0%,北京百灵威科技有限公司)。

大米样品购自唐山市当地超市。

### 1.2 标准溶液的配制

分别称取NIT和OXY 1 mg,使用MeOH将其配制成100 μg/mL的混合标准储备液,将该储备液与BIF标准溶液(100 μg/mL)按照1∶1的体积比配制为50 μg/mL的NIT、OXY和BIF混合标准溶液,4 ℃避光保存。

### 1.3 Fe_3_O_4_@MOF-808吸附剂的构筑

通过溶剂热法制备MOF-808^[15]^。称取174.8 mg ZrCl_4_(0.75 mmol)和52.5 mg H_3_BTC(0.25 mmol)置于烧杯中,依次加入8 mL DMF和8 mL甲酸,超声20 min,将混合溶液移入反应釜中,置于预先加热至120 ℃的烘箱中,反应24 h。反应完毕后于烘箱中自然冷却至室温,离心收集沉淀,采用DMF(30 mL×2)、丙酮(30 mL×2)和MeOH(30 mL×3)依次洗涤,将所得白色粉末于100 ℃真空干燥。

将93.2 mg MOF-808分散于20 mL乙二醇中,超声3 h;取270.0 mg FeCl_3_·6H_2_O和700.0 mg NaOAc·3H_2_O溶解于另一20 mL乙二醇中,超声3 h;将两种溶液混合,超声1 h;将混合溶液移入反应釜,置于预先加热至200 ℃的烘箱中,反应24 h。反应完毕后于烘箱中自然冷却至室温,利用外加磁场分离产物,并用适量乙醇和去离子水清洗产物,直至上清液清澈透明,最后60 ℃真空干燥12 h,得到Fe_3_O_4_@MOF-808吸附剂。

### 1.4 样品制备

准确称取1 g大米样品,充分粉碎,过筛,转移至50 mL离心管中,加入2.5 mL MeOH,超声10 min进行提取,以10000 r/min离心5 min,上清液转移至样品瓶中,超纯水定容至15 mL,待用。

### 1.5 磁性固相萃取流程

称取25 mg Fe_3_O_4_@MOF-808吸附剂,置于处理好的15 mL大米样品溶液中,混匀器以1000 r/min混匀吸附6 min,施加外部磁场将吸附剂与样品溶液分离,弃去上清液。采用0.5 mL MeOH进行洗脱,充分振荡2 min后磁分离,重复2次,合并洗脱溶液,室温下氮吹浓缩至干,MeOH定容至0.5 mL,待测。

### 1.6 色谱分离条件

色谱柱为Agilent XDB C_18_柱(150 mm×4.6 mm, 5 μm);柱温为35 ℃;流动相为(A)H_2_O和(B)MeOH;流速为1 mL/min。洗脱程序为0~5 min, 88%B~89%B,运行时间5 min。进样量为10 μL;检测波长为208 nm。

## 2 结果与讨论

### 2.1 Fe_3_O_4_@MOF-808吸附剂的表征

通过FT-IR对制备的Fe_3_O_4_、MOF-808和Fe_3_O_4_@MOF-808复合材料的基团构成进行表征。如图2a所示,Fe_3_O_4_@MOF-808复合材料在658 cm^-1^和758 cm^-1^处的吸收峰对应MOF-808中Zr-O的外弯曲振动,1380 cm^-1^处的吸收峰归于MOF-808中Zr-O-H基团,1575 cm^-1^处的吸收峰为MOF-808中-COOH的弯曲振动所致^[16]^; 570 cm^-1^处的吸收峰归因于Fe_3_O_4_中Fe-O-Fe的伸缩振动^[17]^。通过XRD对制备的Fe_3_O_4_、MOF-808和Fe_3_O_4_@MOF-808复合材料的晶体结构进行了表征。如图2b所示,Fe_3_O_4_@MOF-808在35.54°、41.12°、50.75°、63.22°、67.89°和74.15°处出现了特征峰,与Fe_3_O_4_相比,特征峰的位置相同,对应Fe_3_O_4_的(311)晶面、(400)晶面、(422)晶面、(440)晶面、(531)晶面和(533)晶面;同时在8.32°和8.69°处出现了与MOF-808谱图对应的特征峰,与文献^[18]^报道一致。

**图2:**
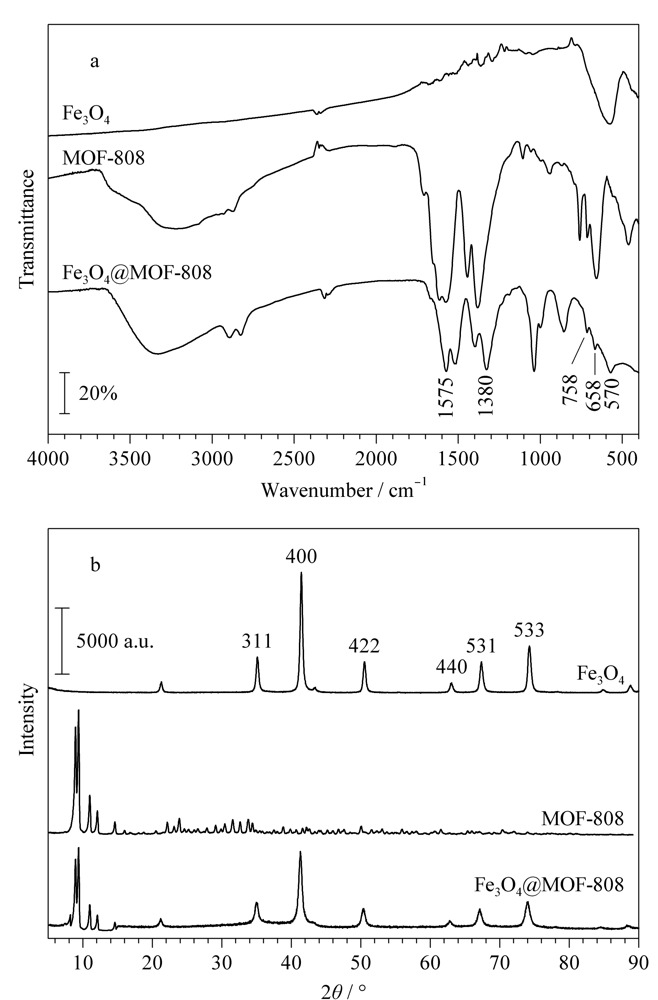
Fe_3_O_4_、MOF-808和Fe_3_O_4_@MOF-808的(a)红外光谱图和(b)X射线衍射图

对制备的Fe_3_O_4_@MOF-808进行扫描电镜分析,观察其形貌。如图3a所示,MOF-808具有规则的八面体形貌,粒径范围在400~500 nm之间,表面较光滑且分布均匀;球形的Fe_3_O_4_纳米颗粒分散于MOF-808表面(见图3b)。采用振动样品磁强计对制备的Fe_3_O_4_和Fe_3_O_4_@MOF-808的磁性进行表征。结果如图4所示,Fe_3_O_4_@MOF-808没有明显的磁滞现象,其饱和磁化强度为40.35 emu/g,虽然较Fe_3_O_4_的饱和磁化强度78.26 emu/g有所下降,但仍然可以满足快速磁分离的要求。

**图3:**
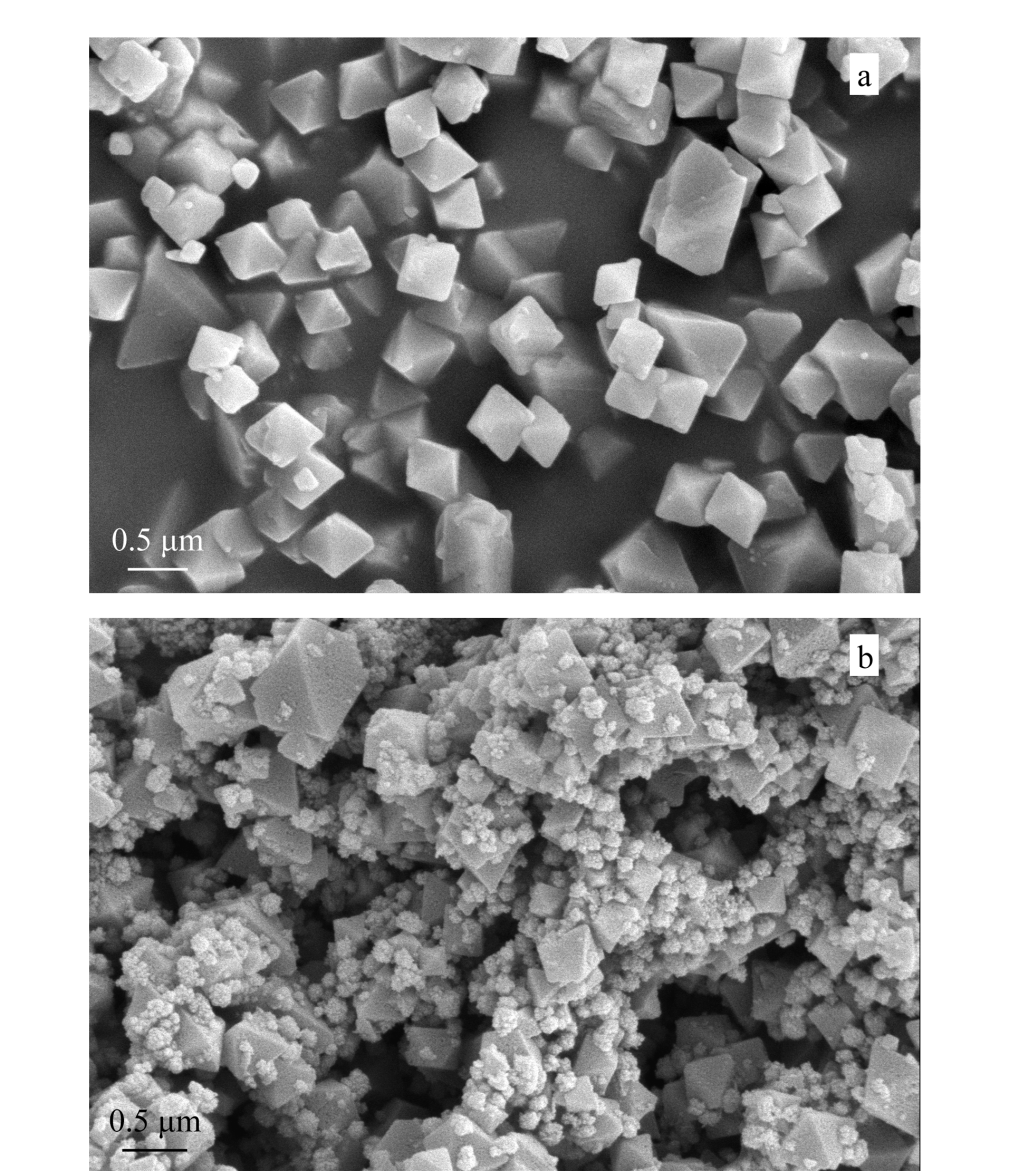
(a)MOF-808和(b)Fe_3_O_4_@MOF-808的SEM图

**图4:**
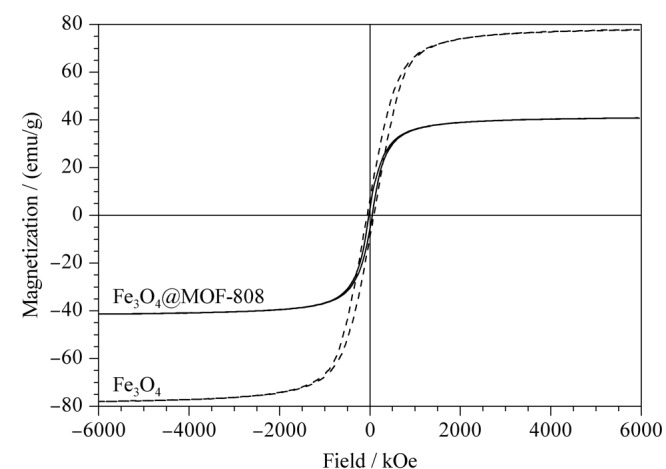
Fe_3_O_4_和Fe_3_O_4_@MOF-808的磁滞曲线图

### 2.2 磁性固相萃取条件的优化

吸附和洗脱过程是影响MSPE效果的关键,为了获得最优的萃取条件,实验对吸附剂用量、吸附时间、洗脱溶剂种类和洗脱体积进行了优化。采用15 mL MeOH-水(1∶5, v/v)混合标准溶液进行实验,3种Des浓度均为65 ng/mL,所有实验平行测定3次。

2.2.1 吸附剂用量和吸附时间

实验分别考察了吸附剂用量为10、15、20、25和30 mg,吸附时间为2、4、6、8和10 min时,3种Des回收率的变化情况。结果如图5a所示,当吸附剂用量为25 mg时,回收率最高。继续增加吸附剂用量,回收率无明显变化。当吸附时间为6 min时,3种Des的回收率均达到95%以上,表明吸附已经达到饱和。因此,实验选择25 mg为最优吸附剂用量,6 min为最优吸附时间。

**图5:**
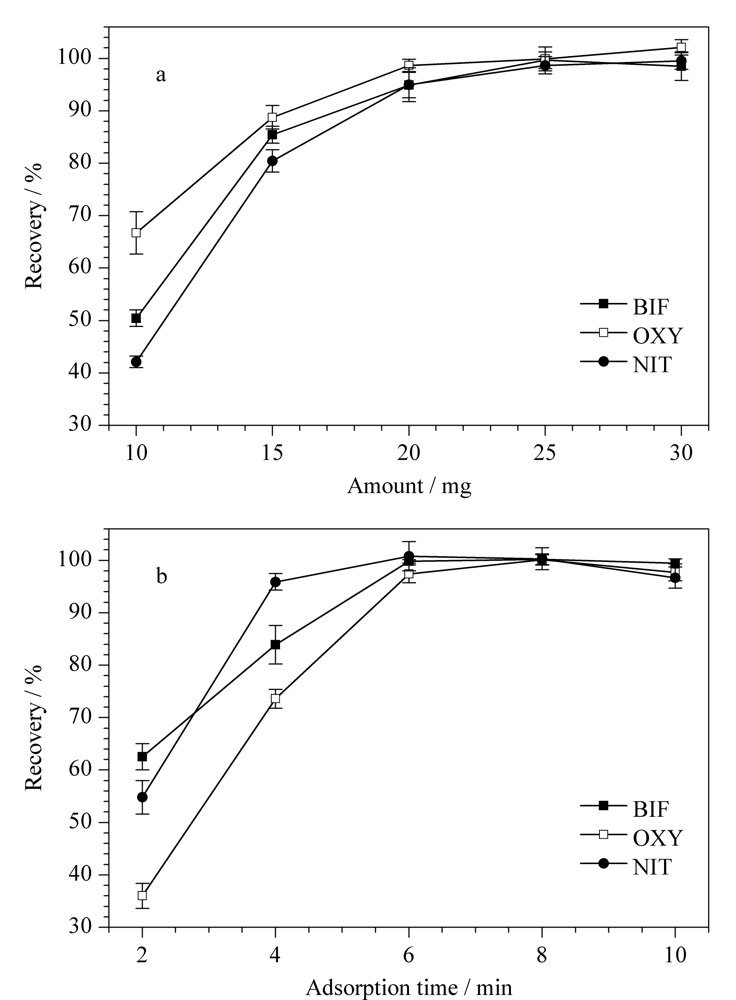
(a)吸附剂用量和(b)吸附时间对Des回收率的影响(*n*=3)

2.2.2 洗脱溶剂和洗脱体积

根据“相似相溶”原理,本实验选取MeOH、ACN和丙酮作为洗脱溶剂。实验固定吸附剂用量为25 mg,吸附时间为6 min,洗脱体积0.5 mL×2,对3种洗脱溶剂进行考察。结果如图6a所示,当洗脱溶剂为MeOH时,回收率最接近100%。实验同时考察了不同洗脱体积(0.5 mL、0.5 mL×2、0.5 mL×3和0.5 mL×4)对Des回收率的影响,结果如图6b所示,当洗脱体积为0.5 mL×2时,3种Des的回收率达到最佳。因此选用0.5 mL×2的MeOH作为最佳洗脱溶剂。

**图6:**
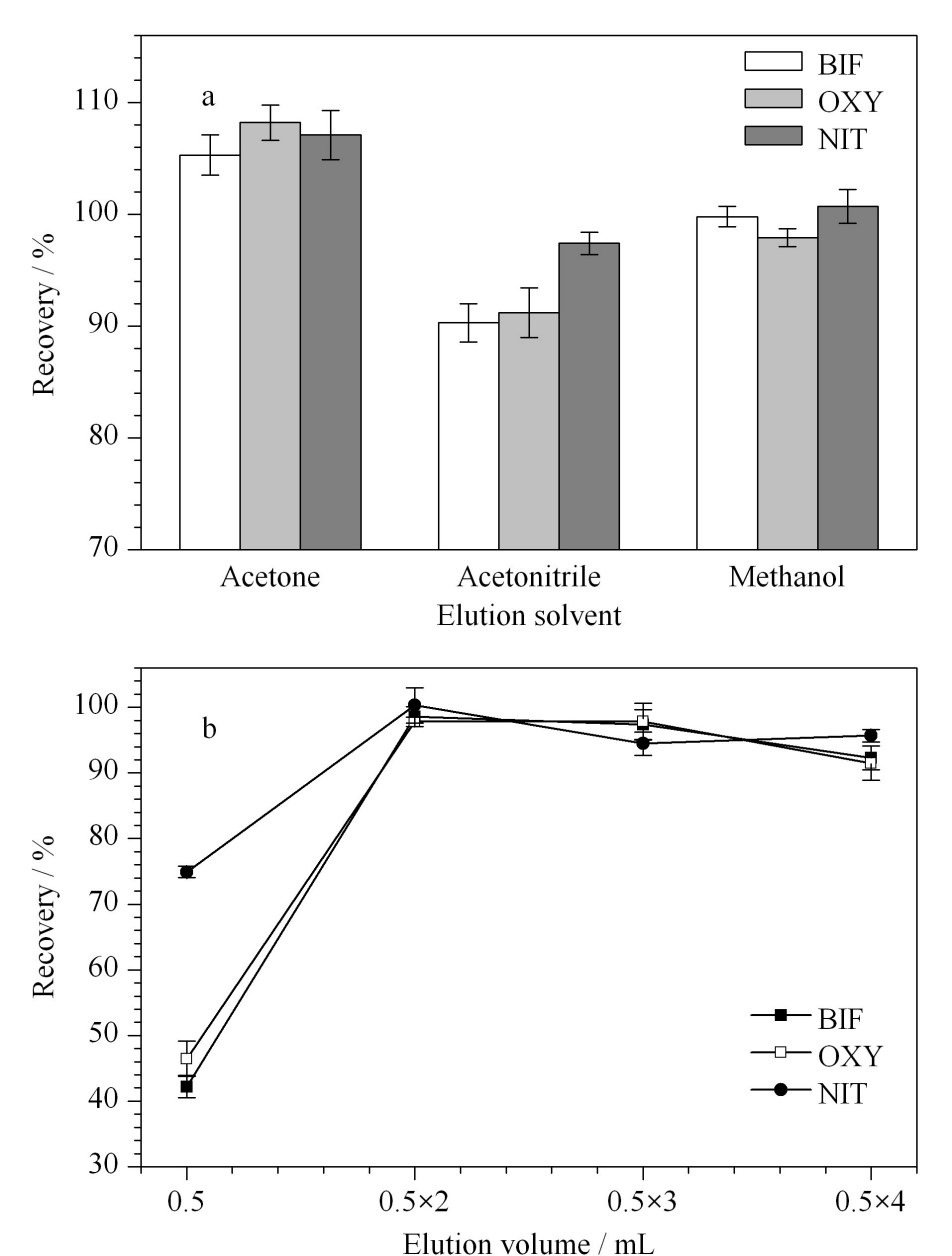
(a)洗脱溶剂种类和(b)洗脱体积对3种Des回收率的影响(*n*=3)

### 2.3 方法验证

2.3.1 线性范围、检出限和定量限

在优化的实验条件下,按照1.4节方法处理空白大米样品,得到空白基质溶液,向其中添加一定量的混合标准溶液配制成质量浓度为2、4、20、40、80、200和300 μg/L的系列基质混合标准溶液,以峰面积(*y*)对除草剂的质量浓度(*x*, μg/L)绘制标准曲线。结果如表1所示,NIT、OXY和BIF在2~300 μg/L范围内线性关系良好,相关系数均大于0.998。以信噪比(*S/N*)=3确定3种除草剂的检出限(LOD),以(*S/N*)=10确定3种目标物的定量限(LOQ),结果见表1。

**表1:** 3种Des的线性范围、检出限和定量限

Analyte	Linear range/ (μg/L)	Regression equation	*r*	LOD/ (μg/kg)	LOQ/ (μg/kg)
BIF	2-300	*y*=56.804*x*-1.0564	0.9992	0.4	1.5
OXY	2-300	*y*=52.783*x*-0.3415	0.9991	0.6	2.0
NIT	2-300	*y*=55.413*x*+0.0566	0.9984	0.6	2.0

*y*: peak area; *x*: mass concentration, μg/L.

2.3.2 回收率和精密度

为了验证方法的准确度和精密度,对空白大米样品进行加标回收试验,3种Des的加标水平分别为5、10和20 μg/kg,每个加标水平平行测定3次。由表2可知,本方法对3种Des的回收率为87.3%~96.7%,日内和日间精密度(*n*=3)分别为2.9%~8.2%和4.2%~10.8%,表明本方法的准确度和精密度良好,能够满足分析要求。

**表2:** 本方法的回收率和精密度(*n*=3)

Analyte	Spiked/ (μg/kg)	Recovery/ %	RSDs/%		
			Intra-day	Inter-day	
BIF	5	89.8	5.8	9.1	
	10	93.1	6.2	8.6	
	20	96.7	4.1	7.5	
OXY	5	91.6	7.4	9.3	
	10	93.5	3.6	4.2	
	20	94.5	2.9	5.8	
NIT	5	87.3	8.2	10.8	
	10	91.4	6.5	9.3	
	20	95.7	4.2	6.7	

### 2.4 实际样品分析

采用最佳的实验条件(25 mg吸附剂吸附6 min),吸附剂与目标物可达到吸附平衡,选择0.5 mL×2的MeOH作为洗脱溶剂,可以将目标物从吸附剂上完全洗脱,结合高效液相色谱法,测定10个大米样品中3种Des,测定结果见表3。所有样品中均未检出这3种Des。通过对每种样品进行加标回收试验证实方法的准确性,结果显示,实际样品中3种分析物的加标回收率(加标水平为10 μg/kg)为85.7%~95.4%, RSD≤11.3%,满足实际样品的分析要求。

**表3:** 大米样品中3种Des的分析结果及加标回收率

Sample	Analyte	Found/(μg/kg)	Recovery/%	RSD/% (*n*=3)
1	BIF	N. D.	87.6	6.2
	OXY	N. D.	90.3	4.1
	NIT	N. D.	88.6	9.3
2	BIF	N. D.	89.3	7.2
	OXY	N. D.	92.5	4.6
	NIT	N. D.	90.4	5.8
3	BIF	N. D.	91.3	2.9
	OXY	N. D.	93.7	3.1
	NIT	N. D.	90.4	5.4
4	BIF	N. D.	88.8	10.1
	OXY	N. D.	92.5	6.5
	NIT	N. D.	91.7	5.7
5	BIF	N. D.	92.6	1.8
	OXY	N. D.	89.7	3.5
	NIT	N. D.	95.4	4.2
6	BIF	N. D.	91.7	4.8
	OXY	N. D.	91.3	6.9
	NIT	N. D.	90.2	10.5
7	BIF	N. D.	90.5	9.1
	OXY	N. D.	92.5	5.6
	NIT	N. D.	89.1	1.8
8	BIF	N. D.	85.7	2.7
	OXY	N. D.	91.3	3.4
	NIT	N. D.	88.6	8.3
9	BIF	N. D.	94.5	6.2
	OXY	N. D.	92.6	3.0
	NIT	N. D.	89.6	7.7
10	BIF	N. D.	92.5	3.9
	OXY	N. D.	90.6	4.5
	NIT	N. D.	91.3	11.3

N. D.: not detected. Added level: 10 μg/kg.

在最优条件下,将3种Des按照20 μg/kg水平加入大米样品,结合HPLC-UV进行分析。图7b为经过粉碎过筛,加入MeOH提取处理的大米样品直接进样的分析结果,杂质峰明显,3种Des均未检出;而经过本方法处理后,结果如图7c所示,杂质峰降低,在对应出峰位置均检出样品峰,说明本方法能够有效富集净化大米样品中的3种Des。在最优条件下,通过对比最终定容的甲醇溶液中3种Des的浓度与大米样品溶液中原始目标物浓度之比,计算本方法对3种Des的富集因子,结果在25~29之间。

**图7:**
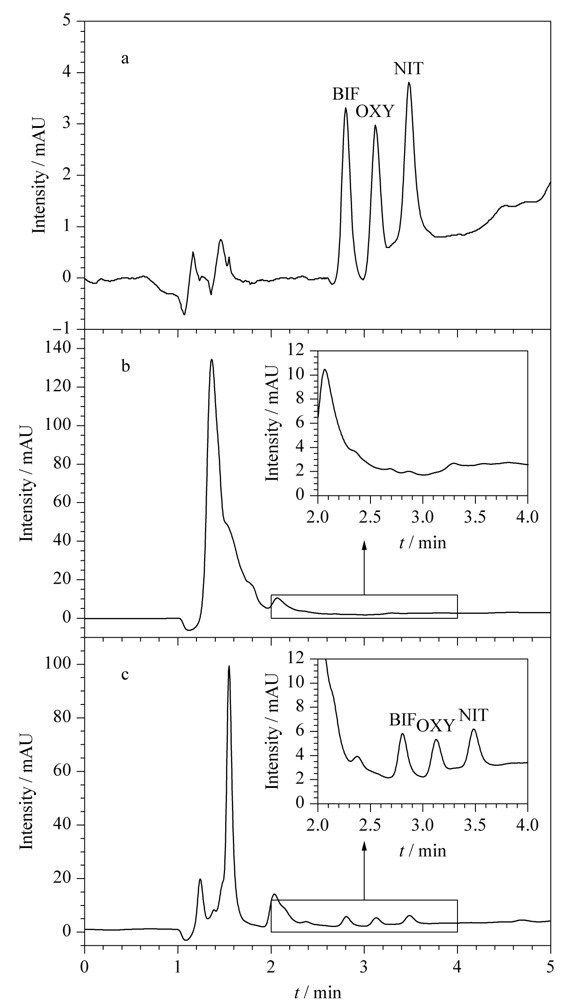
(a)Des标准溶液以及加标大米样品(20 μg/kg)(b)直接进样和(c)经本方法前处理后进样的色谱图

### 2.5 与文献方法对比

将本方法与国家标准方法以及相关文献报道方法进行对比(见表4)。结果显示,本方法的检出限为0.4~0.6 μg/kg,优于国家标准方法,且与其他分析方法相比检出限和回收率相当。在操作时长和简便性方面,本方法使用25 mg吸附剂仅需6 min即可完成对大米样品中Des的吸附,MSPE无需额外的离心、淋洗等步骤,操作简单。

**表4:** 本方法与国家标准方法及文献方法对Des分析结果的对比

Pretreatment method	Instrument	Sample	Extraction time/min	LOD/(μg/kg)		Recovery/%		Reference
QuEChERS	LC-MS/MS	spinach		1	-4	76-	114	[19]
DSPE	GC-MS	soil	10	3.5	-10.5	76-	111	[20]
ASE-DLLE	GC-MS	soil	14	1.2	-2.6	83.5-	103.3	[20]
SPE	GC-MS	rice		25		70-	107	[21]
MSPE	HPLC-UV	wheat flour	25	0.24	-0.68	88.8-	96.6	[22]
MSPE	HPLC-UV	rice	6	0.4	-0.6	87.3-	96.7	this method

QuEChERS: quick, easy, cheap, effective, rugged and safe; DSPE: dispersive solid phase extraction; ASE-DLLE: accelerate solvent extraction-dispersion liquid-liquid extraction; SPE: solid phase extraction; MSPE: magnetic solid phase extraction.

## 3 结论

本研究采用溶剂热法成功制备了Fe_3_O_4_@MOF-808磁性复合材料,将其用作固相萃取吸附剂,结合HPLC-UV分析了大米样品中的3种Des。该方法操作简单、快速,满足大米样品中3种Des的分析要求,为大米样品中除草剂的残留分析提供了新的选择。
